# Outcomes of Patients With Acute Limb Ischemia in Patients With COVID-19: A Systemic Review and Meta-Analysis

**DOI:** 10.7759/cureus.27370

**Published:** 2022-07-27

**Authors:** Ankit Jain, Abhinaya Reddy, Rajeswari Murugesan, Souradeep Dutta, Uday Kumbhar, Ajay Savlania, Sivaranjit K Vaka

**Affiliations:** 1 Surgery, Jawaharlal Institute of Postgraduate Medical Education and Research, Puducherry, IND; 2 Biostatistics, Jawaharlal Institute of Postgraduate Medical Education and Research, Puducherry, IND; 3 Surgery, Postgraduate Institute of Medical Education and Research, Chandigarh, IND

**Keywords:** covid 19-acute limb ischemia, covid-19 retro, mortality, limb-salvage, covid 19, acute limb ischemia

## Abstract

A systemic review and meta-analysis of perioperative outcomes of acute limb ischemia (ALI) in patients with and without coronavirus disease-2019 (COVID-19) to determine the outcomes of ALI and compare the outcomes in patients with and without COVID-19 infection. A literature search of the Medline, Science Direct and Cochrane Library was performed from inception to July 15, 2021. Studies involving ALI in patients with COVID-19 were searched through three electronic databases. The endpoints include limb salvage, re-thrombosis, and mortality, and these outcomes were compared between patients with and without COVID-19 infection and type of management. The primary outcome was early limb salvage (till the patient was discharged from the hospital). The other outcomes assessed were re-thrombosis and mortality. These outcomes were compared between patients with and without COVID-19 infection and the type of management. Pooled estimates were presented as odds ratios (ORs) using a random or fixed effect model based on the results of the chi-square test and calculation of I^2^. Comparing the ALI outcomes in patients with and without COVID-19 infection, there was no significant difference in limb salvage rate (OR=0.26, 95% CI:0.02-3.09), but there was a significantly higher re-thrombosis (OR=2.65, 95% CI:1.34-5.23) and mortality rate (OR=4.71, 95% CI:1.11-19.99) in patients with COVID-19 infection. On comparing outcomes based on management, intervention group, and anticoagulant alone group, no significant difference was noted concerning limb salvage (OR=1.40, 95% CI:0.27-7.13) and mortality rates (OR=0.2, 95% CI:0.04-1.07). This meta-analysis demonstrates a higher re-thrombosis and mortality in ALI patients with COVID-19 infection when compared to patients without COVID-19 but with similar limb salvage.

## Introduction and background

Acute limb ischemia (ALI), sudden disruption of blood flow to the arm and leg, is a vascular emergency. It can occur due to various causes such as cardiovascular emboli, thrombotic occlusion in the background of atherosclerosis or previous vascular reconstruction, vascular injury following a road traffic accident, and in patients with hypercoagulable states such as factor V mutation or protein C deficiency resulting in spontaneous thrombosis [1]. ALI secondary to the hypercoagulable condition can be inherited or acquired and is relatively uncommon. Moreover, hypercoagulable states are prone to recurrent arterial thrombosis [2]. Recently, a pandemic caused by coronavirus disease 2019 (COVID-19) has emerged as an important cause of hypercoagulability, leading to ALI.

Severe acute respiratory syndrome coronavirus 2 (SARS-CoV-2) has been identified as the causative organism of COVID-19. Although morbidity and mortality of COVID-19 are primarily attributed to acute respiratory distress syndrome and end-organ failure, thromboembolic complications, including myocardial infarction, pulmonary embolism, and ischemic stroke, are a significant cause of disability and death in these patients [3]. Venous and arterial thromboembolism associated with COVID-19 coagulopathy is one of the disease’s sequelae associated with poor prognosis [4].

As the pandemic evolved, there has been an unexpected increase in the number of patients with thromboembolic complications, including ALI [5]. However, the data on this was restricted to case reports and a few case series only. While some studies showed comparable results [6], other studies showed the poor outcome of ALI patients suffering from COVID-19 compared to COVID-19 negative patients [7-9]. A systematic review done by Putko et al. concluded an increased risk of limb or digital ischemia in patients with COVID-19 [10]. However, there was no clear data on treatment outcomes as, by the time his review was published, available literature was limited to novel case reports and one case series [10]. After that, numerous studies have been published on COVID-19 in ALI, prompting us to re-evaluate the literature on COVID-19 with ALI. Therefore, this systemic review and meta-analysis were performed to update the evidence in the literature on the outcomes of COVID-19 patients with ALI.

## Review

Methodology

A literature search of the Medline, Science Direct and Cochrane Library was performed before July 15th, 2021. Search terms and boolean operators were: “COVID AND Ischemia” and “SARS-CoV-2 AND Ischemia”. Only articles for which full text was available and in English were included. This meta-analysis is reported in accordance with the Preferred Reporting Items for Systemic Reviews and Meta-analysis (PRISMA) guidelines [11].

Inclusion and Exclusion Criteria

Trials were eligible for inclusion if they fulfilled all the following criteria: (1) randomized control trials, observational (prospective/retrospective) studies, (2) patients diagnosed with SARS-CoV-2, (3) patients with limb ischemia (upper or lower limb) due to arterial thromboembolism, and (4) reported treatment outcomes. The exclusion criteria were: (1) review articles, case reports, case series (articles including ≤ five patients), and conference abstracts; and (2) studies not published in English.

Outcome Variables

The primary outcome was early limb salvage (till the patient was discharged from the hospital). For patients whose status of the limb was not mentioned and expired during the intra-hospital stay, it was taken as limb loss. The secondary outcome was mortality and re-thrombosis rate. Patients were divided into intervention and anticoagulant-only groups based on the intervention method. The intervention group includes patients managed by open surgery (surgical thrombo-embolectomy/surgical bypass/endarterectomy/hybrid procedures), percutaneous endovascular (percutaneous transluminal angioplasty/thrombolysis/mechanical thrombectomy) with or without peri-operative anticoagulants.

Data Extraction

Two independent investigators (AJ and RA) assessed the titles and abstracts separately for relevancy. The abstracts shortlisted were saved for full manuscript review. Articles meeting eligibility criteria were included for the complete analysis, and their references were also reviewed for any eligible study. The following data were independently extracted from each of the included studies by two investigators (AJ and RA): published journal, first author names, published year, study design, sample size, mean age, gender, COVID-19 status, limb involvement (upper/lower limb), treatment received (anticoagulation/surgical/endovascular/hybrid revascularisation). The endpoints included early limb salvage, re-thrombosis rate, and mortality. The disagreement was resolved by consulting the reviewer (UK).

Quality Assessment

Two investigators independently assessed the quality of the studies according to the Newcastle-Ottawa scale [12]. The consensus was reached by the two reviewers (AJ and RA) when there was a difference in opinion on an item. If no consensus was reached, the independent opinion of a third reviewer was decisive (UK).

Data-analysis and Statistical Methods

The meta-analysis was conducted using R-Programming. The odds ratio (OR) with 95% CI was calculated as the summary statistics for dichotomous data. Statistical heterogeneity was assessed using the chi-square test (P-value) and I^2^; if P was >0.1 and I^2^ <50%, the fixed-effects model was used; otherwise, the random-effects model was used to analyze.

Results

A total of 2932 potentially relevant references were found. After the removal of duplicates, abstracts were shortlisted for review. Subsequently, 19 studies were finalized for full-text review, and finally, 11 were included (Table 1).

**Table 1:** The characteristics of studies included in the meta-analysis. IQR: interquartile range.

Author, year	Journal	Country	Study design		Sample size	Gender (M/F)	Mean/median age (years)	Treatment	Primary outcome
Bozzani et al. [4], 2020	Surgery	Italy	Retrospective cohort study	COVID	6	4/2	71 (IQR 49-83)	6 Revascularization	Post-operative mortality and amputation rate
Bellosta et al. [5], 2020	Journal of vascular surgery	Italy	Prospective cohort study	COVID	20	18/2	75 ± 9	3 Medical, 17 revascularization	Successful revascularization, early (<30 days) and late survival, and limb salvage
Mascia` et al. [6], 2020	Annals of vascular surgery	Italy	Prospective cohort study	COVID	16	-	-	15 Revascularization, 1 primary amputation	Limb salvage
				Non-COVID	21	-	-	16 Revascularization, 5 primary amputation	Limb salvage
Bellosta et al. [7], 2021	European society for vascular surgery	Italy	Multicentric, retrospective, observational cohort study	COVID	55	-	73 ± 12	55 Revascularization	Freedom from in-hospital death
				Non-COVID	89	-	72 ± 12	89 Revascularization	
Goldman et al. [8], 2020	Radiology	USA	Retrospective cohort study	COVID	16	9/7	70 ± 14	-	Clot burden in patients with COVID-19
				Non-COVID	32	16/16	71 ± 15	-	
Yesilkaya et al. [9], 2021	Annals of vascular surgery	Turkey	Retrospective cohort study	COVID	11	5/6	71.2 ± 13.9	Revascularization	Clinical characteristics of patients histopathological characteristics of thrombo-embolic material
				Non-COVID	10	6/4	61.8 ± 15.5	Revascularization	
Indes et al. [13], 2020	Journal of vascular surgery	USA	Retrospective cohort study	COVID	13	9/4	63.3 (54.5-65.5)	3 Conservative, 6 medical, 4 Revascularization	Identify potential risk factors for arterial thromboembolic disease
Topcu et al. [14], 2021	Annals of Vascular Surgery	Turkey	Retrospective cohort study	COVID	6	5/1	62 (IQR, 59-64.3)	3 Medical, 3 revascularization	Outcomes of COVID-19 patients with ALI
Ilonzo et al. [15], 2020	Journal of vascular surgery	USA	Retrospective cohort study	COVID	16	8/8	63.3	3 Medical, 13 revascularization	Mortality secondary: primary patency and morbidity
Sanchez et al. [16], 2021	Annals of vascular surgery	Peru	Retrospective cohort study (multicentric)	COVID	30	23/7	60 ± 15	3 Medical, 28 revascularization	Clinical and surgical characteristics
Al-zoubi et al. [17], 2021	International journal of medicine	Jordan	Retrospective cohort study	COVID	7	5/2	65.56 ± 1.13	5 Medical, 2 revascularization	Demography and outcomes

**Figure 1:**
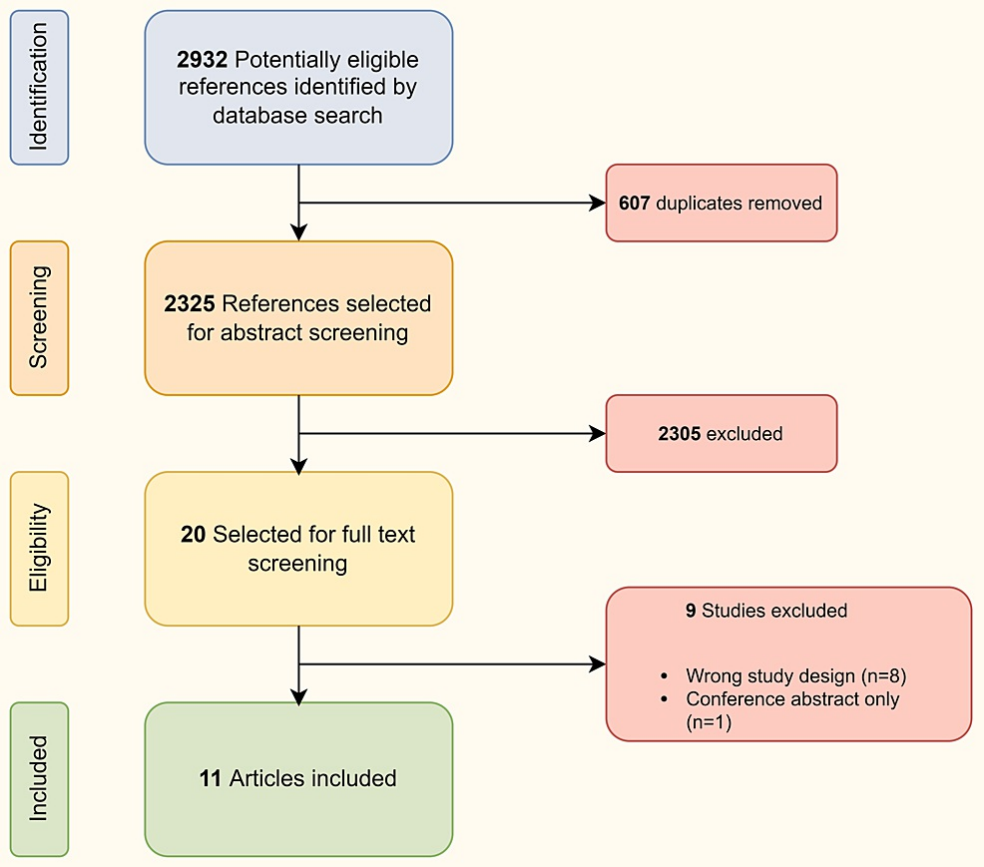
PRISMA flow diagram showing the identification of eligible and participating studies. PRISMA: preferred reporting items for systemic reviews and meta-analysis.

The literature search process is illustrated as a flow diagram in Figure 1.

The survey by Bellosta et al. [7] published in 2021 included three [4-6] other previously published articles. However, the data on mortality and limb salvage was not available for all the patients; therefore, it was decided to include the three original articles [4-6], and data from Bellosta et al. [7] was used only to pool the revascularization failure rate.

Study Quality

The quality of studies is shown in Table 2. Four studies [6,9,10,13] were of good quality, and six studies [4,5,14-17] were of poor quality.

**Table 2:** Study quality. Good quality: Three or four stars in the selection domain AND one or two stars in the comparability domain AND two or three stars in the outcome/exposure domain. Fair quality: Two stars in selection domain AND one or two stars in comparability domain AND two or three stars in outcome/exposure domain. Poor quality: Zero or one star in selection domain OR zero stars in comparability domain OR zero or one star in outcome/exposure domain.

Author	Patient selection				Comparability of cohorts	Outcomes			Quality of study
	Representatives of exposed cohort	Selection of non-exposed cohort	Ascertainment of exposure	Outcome of interest not at present of the study		Assessment of outcome	Adequate follow-up	Adequacy of follow-up	
Bozzani et al. [4]	*	-	*	*	-	*	-	-	Poor
Bellosta et al. [5]	*	-	*	*	-	*	*	*	Poor
Mascia et al. [6]	*	*	*	*	*	-	*	*	Good
Goldman et al. [8]	*	*	*	*	**	*	-	*	Good
Yesilkaya et al. [9]	-	*	*	*	**	*	-	*	Good
Indes et al. [13]	*	*	*	*	**	*	*	*	Good
Topcu et al. [14]	*	-	*	*	-	*	*	*	Poor
Ilonzo et al. [15]	*	-	*	*	-	*	-	*	Poor
Sanchez et al. [16]	*	-	*	*	-	*	*	*	Poor
Al-zoubi et al. [17]	*	-	*	-	-	*	-	*	Poor

*Limb Salvage* 

**Figure 2:**
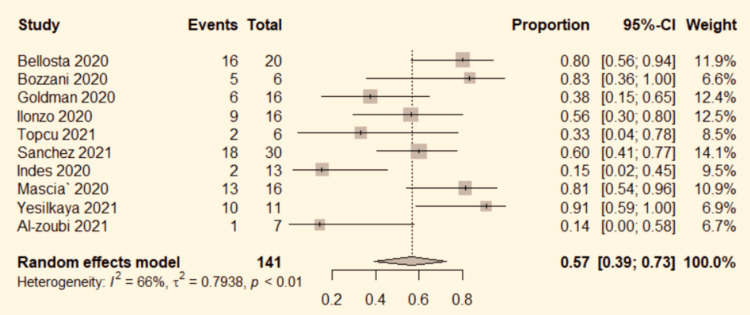
Forest plot for limb salvage in COVID-19 positive ALI patients. ALI: acute limb ischemia, CI: confidence interval, COVID-19: coronavirus disease 2019, OR: odds ratio. Bozzani et al. [4], Bellosta et al. [5], Mascia et al. [6], Goldman et al. [8], Yesilkaya et al. [9], Indes et al. [13], Topcu et al. [14], Ilonzo et al. [15], Sanchez et al. [16], and Al-zoubi et al. [17].

Ten studies [4-6,8,9,13-17] mentioned the limb salvage rate. The data were pooled to do the analysis. The studies had significant heterogeneity (P<0.01, I^2^=66%). Therefore, the random-effects model was used. The pooled result showed a 57% limb salvage rate (95% CI: 39%-73%; Figure 2) in COVID-19 patients with ALI.

**Figure 3:**
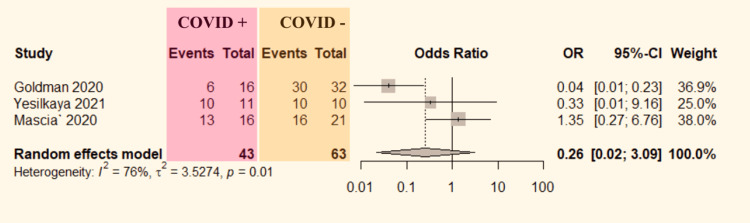
Forest plot comparing limb salvage in COVID-19 positive versus COVID-19 negative ALI patients. ALI: acute limb ischemia, CI: confidence interval, COVID-19: coronavirus disease 2019, OR: odds ratio. Mascia et al. [6], Goldman et al. [8], and Yesilkaya et al. [9].

Three [6,8,9] studies mentioned limb salvage rate comparison data between ALI patients with and without COVID-19. The data were pooled to do the analysis. The studies showed significant heterogeneity (P=0.01, I^2^=76%). Therefore, the random-effect model was used. There was a similar probability of limb salvage (OR=0.26, 95% CI: 0.02-3.09; Figure 3) in ALI patients with or without COVID-19.

Re-thrombosis

**Figure 4:**
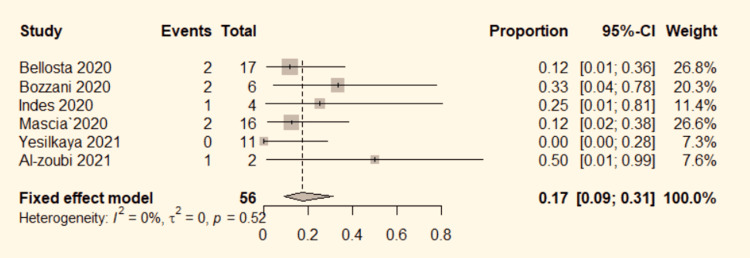
Forest plot for re-thrombosis in COVID-19 positive ALI patients. ALI: acute limb ischemia, CI: confidence interval, COVID-19: coronavirus disease 2019, OR: odds ratio. Bozzani et al. [4], Bellosta et al. [5], Mascia et al. [6], Yesilkaya et al. [9], Indes et al. [13], and Al-zoubi et al. [17].

Six studies [4-6,9,13,17] mentioned the re-thrombosis rate in the revascularized limb in ALI patients with COVID-19. The data were pooled to do the analysis. No significant heterogeneity was detected between the studies (P=0.52, I^2^=0%). Therefore, the fixed-effect model was used. The pooled results show a 17% overall re-thrombosis rate (95% CI: 9%-31%; Figure 4).

**Figure 5:**
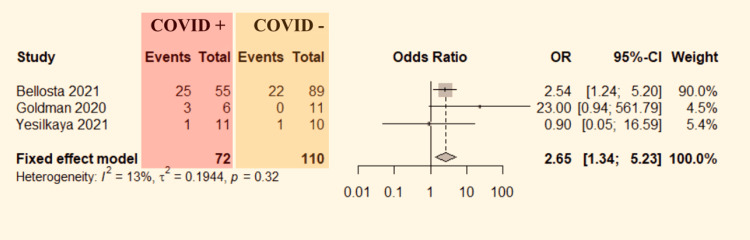
Forest plot for comparison of re-thrombosis in COVID-19 positive versus COVID-19 negative ALI patients. ALI: Acute limb ischemia, CI: confidence interval, COVID-19: coronavirus disease 2019, OR: odds ratio. Bellosta et al. [7], Goldman et al. [8], and Yesilkaya et al. [9].

Three studies [7-9] mentioned the re-thrombosis rate comparison data between ALI patients with and without COVID-19. The data were pooled to do the analysis. No significant heterogeneity was detected between the studies (P=0.32, I^2^=13%). Therefore, the fixed-effect model was used. The pooled result shows a significantly higher probability of re-thrombosis in a revascularized limb (OR=2.65, 95% CI: 1.34-5.23; Figure 5) of ALI patients who were COVID-19 positive.

Mortality

**Figure 6:**
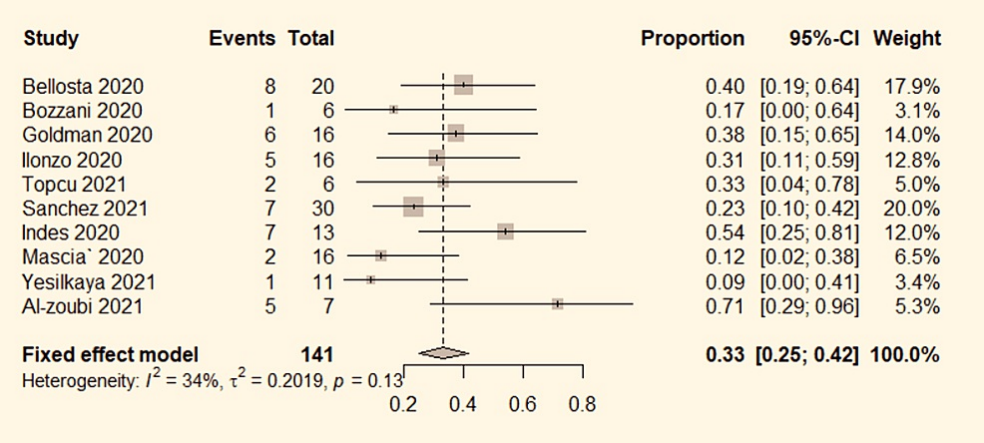
Forest plot for mortality in COVID-19 positive ALI patients. ALI: acute limb ischemia, CI: confidence interval, COVID-19: coronavirus disease 2019, OR: odds ratio. Bozzani et al. [4], Bellosta et al. [5], Mascia et al. [6], Goldman et al. [8], Yesilkaya et al. [9], Indes et al. [13], Topcu et al. [14], Ilonzo et al. [15], Sanchez et al. [16], and Al-zoubi et al. [17].

Ten studies [4-6,8,9,13-17] mentioned the overall mortality rate in COVID-19 patients with ALI. The data were pooled to do the analysis. No significant heterogeneity was detected between the studies (P=0.13, I^2^=34%). Therefore, the fixed-effect model was used. The pooled results show a 33% overall mortality rate (95% CI:25%-42%; Figure 6) in ALI patients who were COVID positive.

**Figure 7:**
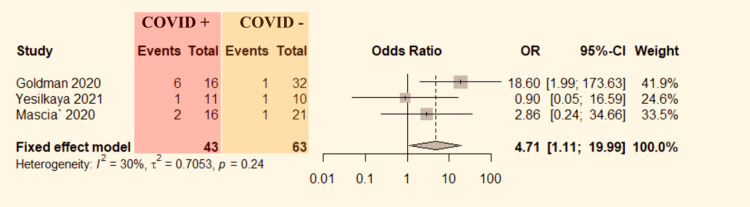
Forest plot comparing mortality in COVID-19 positive vs COVID-19 negative ALI patients. ALI: acute limb ischemia, CI: confidence interval, COVID-19: coronavirus disease 2019, OR: odds ratio. Mascia et al. [6], Goldman et al. [8], and Yesilkaya et al. [9].

Three studies [6,8,9] mentioned mortality rate comparison data between ALI patients with and without COVID-19. The data were pooled to do the analysis. No significant heterogeneity was detected between the studies (P=0.24, I^2^=30%). Therefore, the fixed-effect model was used. The pooled result shows a significantly higher probability of mortality in COVID-19 patients compared to non-COVID-19 patients with ALI (OR=4.71, 95% CI:1.11-19.99; Figure 7).

Intervention Versus Anticoagulant Alone in COVID-19 Positive Patients

**Figure 8:**
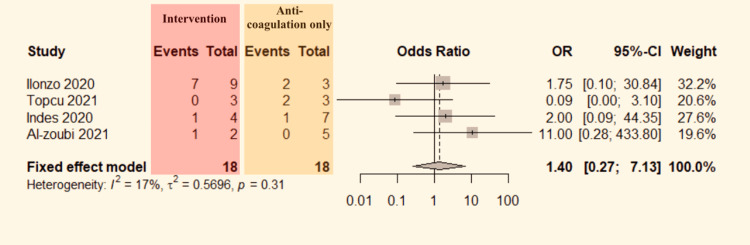
Forest plot comparing limb salvage in COVID-19 positive ALI patients who underwent intervention versus anticoagulation alone. CI: confidence interval, OR: odds ratio. Indes et al. [13], Topcu et al. [14], Ilonzo et al. [15], and Al-zoubi et al. [17].

Limb Salvage: Data regarding limb salvage between intervention and anticoagulant alone can be extracted from four studies [13-15,17]. The data were pooled to do the analysis. No significant heterogeneity was detected between the studies (P=0.31, I^2^=17%). Therefore, the fixed-effect model was used. The pooled result shows no significant difference in the effect of the treatment method on the limb salvage rate (OR=1.40, 95% CI:0.27-7.13; Figure 8) of ALI patients who were COVID positive.

**Figure 9:**
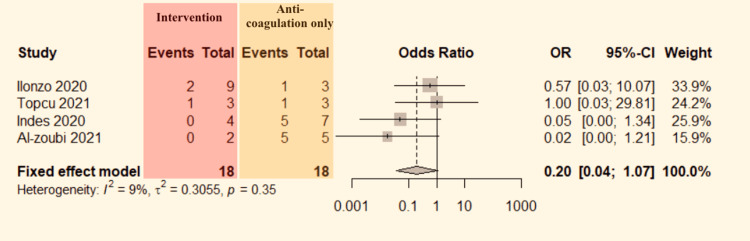
Forest plot comparing mortality in COVID-19 positive ALI patients who underwent intervention versus anticoagulation alone. CI: confidence interval, OR: odds ratio. Indes et al. [13], Topcu et al. [14], Ilonzo et al. [15], and Al-zoubi et al. [17].

Mortality: Data regarding mortality between intervention and anticoagulant alone can be extracted from four studies [13-15,17]. The data were pooled to do the analysis. No significant heterogeneity was detected between the studies (P=0.35, I^2^=9%). Therefore, the fixed-effect model was used. The pooled result shows no significant difference in mortality (OR=0.20, 95% CI:0.04-1.07; Figure 9) between the groups.

Discussion

The critical findings of our systemic review and meta-analysis were that COVID-19 patients affected with ALI are associated with a low limb salvage rate, a high rate of re-thrombosis, and mortality. Although no significant difference was noted in limb salvage rates in patients of ALI with and without COVID-19 infection, COVID-19 patients with ALI had significantly higher re-thrombosis rates following revascularization and mortality. No significant difference was noted in limb salvage and mortality rates between the intervention and anticoagulant alone groups.

The studies on ALI in patients with COVID-19 were limited to prospective and retrospective cohort studies apart from case series and case reports because of the unexpected, concomitant, and rare occurrence of ALI and COVID-19, making controlled trials impractical. Even though the exact mechanism of the prothrombotic state in COVID-19 is not known, various theories proposed are endothelial dysfunction, activation of the immune system and complement pathway, impairment of coagulation, hypoxia induces transformation of endothelium into a prothrombotic phenotype, and other traditional risk factors immobilization may predispose the patient to thromboembolic complications [18-20].

The reported rate of thromboembolic complications in COVID-19 varies widely from 20% to 70% [21,22]. This wide variation in the incidence may be attributed to the inclusion of COVID-19 patients with different severity in the studies, non-uniformity in screening and diagnostic methods, and varied protocols of using anticoagulants across the studies. The coagulopathy due to COVID-19 has been preferentially associated with venous thromboembolism, while arterial thromboembolism (ATE) was noted less frequently [20,22]. The association of arterial thrombosis with COVID-19 was first reported by Klok et al. [23], and since then, several reports of arterial thrombosis involving various arterial segments manifesting as ischemic strokes, coronary artery disease, aortic thrombus, ALI, as well as arterial thrombi in unusual sites resulting in acute mesenteric ischemia and splenic infarct have been reported [16].

A 3-15% in-hospital incidence of ALI in COVID-19 patients has been reported, which is much higher than that in the general population (10-15 per 1,00,000 population) [24,25]. Moreover, Goldman et al. studied the distribution of thrombus in ALI patients with COVID-19 and with matched ALI patients without COVID-19 and concluded that COVID-19 patients had significantly more thrombus burden, involving proximal large and medium vessels [8], and vessels often without underlying atherosclerotic changes [13]. The reason for this increase in ALI in COVID-19 is the underlying COVID-induced prothrombotic state [18,19]. Even though there is an association between ATE and COVID-19 infection, there appears to be no correlation between the severity of the COVID-19 and the risk of ATE, as ALI is seen in patients with mild symptoms. Even in some patients presenting with ALI later on, routine screening identified COVID-19 infection [5,15,24,26].

The limb salvage rates in our review were 57% in patients with COVID-19, which is lower than the limb salvage rates seen in the literature of 70-90%, depending on the etiology of ALI [1,27,28]. This dismal outcome may be attributed to the delayed presentation due to the difficulty in accessing proper health care facilities during the COVID-19 pandemic [5] and thrombophilia caused by COVID-19. Thrombophilia, either acquired or inherited, is associated with poor limb outcomes [2]. Moreover, venous thromboembolism is seen commonly in thrombophilia, while ATE is rare. Therefore, literature on the outcomes following revascularization in thrombophilia in ALI is limited due to the rarity. Patients with cancer and cancer chemotherapy agents, a known risk factor for thrombophilia, are at an increased risk of thromboembolic complications. Tsang et al. noted a 37% limb loss in patients with ALI following revascularization for ALI in cancer patients developing ALI, which is similar to our study [29]. Even though limb salvage was not significantly different compared to ALI in non-COVID-19 patients, this may be due to very few studies with comparison groups, including a small number of patients, and may also be attributed to the heterogeneity of the studies. Moreover, thrombophilia is also associated with increased re-thrombosis and limb loss in patients following bypass for peripheral arterial occlusive disease [30]. Ni et al. noted a significantly high re-thrombosis and major amputations (29% and 11%) in patients with thrombophilia [31]. Therefore, the high rate of re-thrombosis seen in our study who underwent re-vascularisation could be attributed to the prothrombotic state in COVID-19.

A high mortality rate (33%) was noted in our review in patients with ALI with COVID-19 infection. The mortality rates in COVID-19 infections vary from 5% to 54.6%, depending on the severity of the infection [32]. Along with severity, several factors affect the mortality in COVID-19 infection like older age, male sex, a high fraction of inspired oxygen, high positive end-expiratory pressure, intensive care admission, thrombophilia, and history of chronic obstructive pulmonary disease, hypercholesterolemia, and type 2 diabetes [33]. Among these, thrombophilia is an important determinant of mortality. Even in a patient with thrombophilia, patients with ALI were associated with a ten times higher risk of mortality when compared to pulmonary embolism [34]. 

We intended to assess limb and patient outcomes between different methods of intervention. However, except for one study by Ilonzo et al. in which five patients were managed by an endovascular approach, one patient required surgical thrombectomy [15]. In contrast, two patients expired, and limbs were salvaged in two patients. Hence, in all other studies, surgical thrombo-embolectomy was the mainstay of treatment; hence, we could not compare the results and conclude a better management method for these patients. In all studies, patients who underwent revascularisation received perioperative therapeutic doses of anticoagulants.

The critical aspects of future research are: (1) as planning a controlled trial is impractical in this group of patients, a good quality prospective study with predefined criteria for diagnosis, intervention, and outcomes of ALI in patients with and without COVID-19 is required, (2) studies on endovascular methods of intervention like thrombolysis or percutaneous mechanical thrombectomy may be endeavored to decrease the mortality rates in this group of patients, (3) in all the previous studies, a high rate of re-thrombosis and lower limb salvage was noted despite the patients being on the therapeutic level of anticoagulation. Studies with anticoagulants at levels higher than the therapeutic range might be considered.

The limitations of this review include: (1) significant heterogeneity is seen concerning the outcomes of the studies. This may be due to differences in the presentation time to the onset of symptoms, delay in intervention as different institutional protocols in managing COVID-19 patients, and different intervention methods (one study, four cases hybrid procedure, one study, four cases were handled by endovascular approach), (2) few studies, including a small number of patients, compared ALI patients with and without COVID-19 infection, making the results questionable, (3) even in the studies where comparison was made, most of them used historical data on ALI without COVID-19 as a comparison group, and (4) the majority of our studies were of poor quality; the main reason was that only a few studies had a comparison group.

## Conclusions

There has been a significant increase in patients with thromboembolic complications like ALI during the COVID-19 pandemic. Our meta-analysis demonstrates that ALI patients with COVID-19 have higher re-thrombosis and mortality rates than non-COVID-19 with similar limb salvage. Outcomes were similar irrespective of the method of intervention. Further good-quality studies with pre-and well-defined criteria are needed to reinforce these findings and determine the better management method for these patients for optimal patient outcomes.
